# Ossified Pulps

**Published:** 1872-01

**Authors:** S. P. Cutler


AMERICAN JOURNAL

OF

DENTAL SCIENCE.

Vol. V. THIRD SERIES--JANUARY, 1872. No. 9.

### ARTICLE I.

### *Ossified Pulps.*

BY S. P. CUTLER, M. D., D. D.

Read before the Tennessee Dental Association.

A lady about 35 years old called to have all her upper teeth extracted, so that a dentist in the country where she lived could put her in a full upper set.

The wisdom tooth on the right side was very small and sound, the second molar decayed off, the first molar, two bicuspids, and cuspid quite good with some cavities of decay; the four incisors gone, and three teeth, the right lateral, and two centrals pivoted, the lateral pivot broken and tooth gone, the left lateral and cuspid were nearly off to the gums, the left first bicuspid dead, with a large filling and another cavity; all teeth back on that side decayed off, which I extracted, and advised pivoting the other two, which she consented to.

On drilling the pivot hole in the lateral fang, I found no nerve cavity at all, the ossification complete and hard above the point drilled, no soreness or tenderness of and kind, the cuspid was also ossified one half the depth of the pivot hole, no tenderness whatever.

She had worn those three pivot teeth for over ten years without any trouble whatever, and done most her chewing in front of late years. She was well pleased with my advice and work.

In the above case we learn a valuable practical lesson, that is, that in all such cases where there is ossification without any trouble accompanying the process, we may proceed with our operations whatever they may be with almost perfect confidence of success. Where there is too much waste in the process, creosote and and other palliative treatment will in many instances procure relief from pain or soreness, as preliminary measures to operating.

I have recently adopted the plan, where there is any exposure of nerve and no inflammation, to cap the nerve with a small slice of spunk, one side slightly moistened with creosote and applied directly over the point of exposure, and then filling either with os-artificiel or amalgam, as the case may require. Where I wish to fill with gold, I use os-articiel first, and let it remain for a few weeks or months, then cut away sufficient to form the cavity to my liking, then fill without disturbing the capping and the portion of bone filling immediately over.

I have in this way filled a large number of badly decayed teeth, whether exposure of nerve existed or not, and with more *decided success* than by any other plan I ever practiced.

The spunk serves as a complete non-conductor and at the same time a protection to the nerve and sensitive dentine in the neighborhood, by its peculiar soft and delicate texture and non-destructibility, it being almost pure carbon which makes it, in my opinion, pre-eminently superior to any other substance yet brought into use for like purposes.

Now one of two things always occurs sooner or later where there is nearly or quite an exposure of pulp and the tooth filled, that is, the pulp either ossifies or dies; sometimes even the former condition first takes place to a limited extent, and subsequent death from too much continual shocking by the conductivity of the filling. In cases where the spunk is used for capping, the capping stands between the trembling pulp on the one hand and the shocks of the plug on the other hand.

No possible harm can arise from its presence in the bottom of all large cavities, as it is a palladium of safety to the delicate pulp and exposed ends of fibrils occupying the dentinal tubuli. In consequence of this protectlon, ossification, which must take place sooner or later on the inner walls of the pulp cavity or in the pulp at this point, or both consecutively may be carried on in a more normal or unhurried manner than must otherwise necessarily be the case, and as a consequence, in a large number of cases, the nerve and tooth be saved alive, when under other circumstances it would die and give trouble.

As a general rule, in all cases of exposure, or nearly so, where ossification takes place, the process is commenced immediately under the point of exposure, from the fact that at this point, there has been destroyed and removed a large extent of nerve fibrils that formerly dwelt in the tubuli, where there was a normal balance between the vital fibrils and mineral dentine resulting in a physiological or healthy balance.

Now where so large an extent of both non-vital and vital tissue have been carried away by disease, a corresponding weakening or vital resistance has taken place, which from this very weakening at the decayed point, yields to the more dominant influence of mineralisation by slow, gradual accumulations of lime salts in the pulp, first by stacis in the capillaries, then infiltrations of the liquor sanguinis through the capillary walls into pulp tissue among the nerve fibrils

At first we notice a chalky appearance at that point, of greater or less extent, of a granular mass not yet hardened into secondary dentine but undergoing this process. This can be often seen by the naked eye on removal of such pulps.

Sometimes there is a general white chalky appearance throughout the entire pulp, as if it was injected and distended with some white substance, which on exposure to the air does not shrink away like ordinary pulps into a rinkled and corrugated piece of raw hide, but remains full and plump like a dressed partridge ready for the cook pot.

Some one more incredulous than the balance of us poor mortals, asks "if this is really the case?" I will simply answer him by asking him to just make the experiment for himself and see for himself, as seeing is to know it as positive evidence.

I do not ask the profession to believe all I say in the matter, but let them investigate for themselves and give us their knowledge as data for us to go by; where there is multitude of counsel there is supposed to be safety.

Those chalky pulps make very interesting specimens for the microscope.

After this stuffed appearance of the pulp, there is very little evidence of any red blood circulating in the tissue, though there necessarily must be liquor sanguinis, circulating there until the process of hardening is complete, as this same pulp possesses more or less sensibility, which I know from personal knowledge in a great number of cases.

The nerve fibrils of the pulp, even after the process of hardening is complete, sometimes remains quite sensitive to the touch of an excavator, an example of which I witnessed recently in a very interesting case.

A certain quack doctor had a left anterior lower molar decayed on the posterior proximal side to the nerve cavity. When I saw this case the tooth had given trouble for several nights so as to prevent sleep, though not like common tooth-ache. I found the pulp entirely ossified so far as I drilled, which was down as low as the bottom of the nerve cavity, and solid dentine all the way, with some sensibility on cutting and drilling.

A great number of applications to relieve, were made which had only temporary effect, but subsequently the tooth did get better and was in his mouth the last time I saw him, but not entirely well. Up to that time this tooth had given him no trouble, still the process of ossification was nearly, completed throughout, and the probability is that he may be able to keep the tooth, as there is no soreness on masticating. In this case the pulp near the ends of fangs may be the point of trouble where the fibrils are too much pressed upon by the new process.

Another case: One of our dental students about 20 years old had an upper bicuspid filled with gold, which gave trouble, and he had the filling taken out which gave entire relief. When I saw the tooth, it had a large anterior approximal cavity occupying nearly the whole side of the tooth which would have penetrated the pulp cavity, only that the pulp was ossified and of a dark brown appearance, firm and solid. I believe this tooth had had an amalgam filling in it, and this may account for the discoloration. I advised him to use creosote in the cavity for a time, then cap and fill with os-artificiel and at some future time have it permanently filled.

Many other instructive cases have come under my own observation that I might relate, which are equally instructive in a practical point of view.

Now we want more practical knowledge in this important direction of our profession. What does our actual knowledge amount to, and what data is to day before the profession, that is of any defined and decided practical advantage to us? We are all groping our way in the dark, like the blind leading the blind, with little heed whether we go right or wrong so that we keep going. We all know how to pull out and put in teeth, but we want to know how to avoid this under all circumstances, so long as a tooth has a live pulp in it. After the death of this important tissue, we are then dealing with not exactly dead men's bones, but with dead bones themselves, in other words we have dead cocks in the pit to deal with, and it is an old truism "that a dead lion is not worth a live dog."

One reason why there are not more ossified pulps met with from decays reaching them, I believe to be owing to the fact that decay in teeth is the direct action of an acid, and as the decay approaches the pulp cavity, the dentine at the bottom of decay down to the chamber becomes more or less saturated with acid, and certain portions of the lime salts decomposed with discoloration down to the pulp, with still vital fibrils occupying this portion of affected dentine; I have often witnessed this condition of things.

The acid condition of this portion of dentine we might imagine to be sufficient cause to prevent secondary ossification in a large majority of cases. In fact I believe this tendency to secondary ossification is universal when the decay has approached the chamber, but owing to the presence of acid action in this vicinity the process is either altogether prevented, or at least retarded in a majority of cases. Nodulation, where the pulp has been reached, is a much more common occurrence than is generally supposed. Sometimes exceedingly small nodules may be found, several in the same pulp. Now what I propose, is to neutralize the acid always before attempting to fill, so as to favor this secondary process. This may be done by either carbonate of soda and ammonia, which should be sealed up in the cavity over night, or at least several hours, then dressing with creosote before filling. In this way we have assurance that the diseased dentine, is undoubtedly saturated to a greater or less extent with some organic acid, which has worked the destruction of the tooth so far as the decayed portion is concerned. In this way we may favor this saving process without any hazard whatever, with greater promise of success than from our usual method of filling such teeth without any treatment preceeding the operation.

Another case of nodular dentine. A sister of charity, 60 years or more of age, called and had a solitary left upper molar extracted. This tooth was very loose, still held fast by fleshy attachments. The tooth was sound and of good density, the fangs yellowish translucent, showing structural change in tubuli and slight cementosis.

On splitting it open, I found a black nodule hard and adherent to neck portion of cavity. This is an unusual case.

In this case the nodule was formed during the life of the pulp, as is always the case; and before the process occupied the entire pulp, death overtook the pulp which stopped all further action in this direction. The death of the pulp may have been the result of loosening or some other cause.

I have now in my possession over a hundred teeth with nodular and other forms of ossified pulps.

I meet with teeth almost daily with deep decays and no exposure of pulp, owing to the fact that ossification has taken place under the decayed region; sometimes this is the case in very young persons. In all such cases if there has been no trouble with the tooth recently, there is but little uncertainty in filling such teeth. Such teeth have acted on the defensive successfully against all outward attacks, and with a little assistance on our part, success is, I may say, certain, in at least a large majority of cases; I have seldom failed in such cases. It is an easy matter to determine whether or not there is any ossification of pulp by a tolerable strong magnifyer, the non-fleshy appearance of the pulp beingour best guide in these cases, together with the hardened condition at bottom of cavity.

